# Age-related sex differences in the outcomes of patients with hypertrophic cardiomyopathy

**DOI:** 10.1371/journal.pone.0264580

**Published:** 2022-02-25

**Authors:** Hyun-Jung Lee, Hyung-Kwan Kim, Sang Chol Lee, Steve R. Ommen, Jihoon Kim, Jun-Bean Park, You-Jung Choi, Seung-Pyo Lee, Sung-A. Chang, Yong-Jin Kim

**Affiliations:** 1 Cardiovascular Center, Seoul National University Hospital, Seoul National University School of Medicine, Seoul, Korea; 2 Cardiovascular Imaging Center, Heart Vascular Stroke Institute, Samsung Medical Center, Sungkyunkwan University School of Medicine, Seoul, Korea; 3 Division of Cardiovascular Diseases, Mayo Clinic College of Medicine, Rochester, Minnesota, United States of America; Karolinska Institutet, SWEDEN

## Abstract

**Background:**

We aimed to clarify the sex differences in various cardiovascular and non-cardiovascular outcomes, and to investigate whether sex differences in outcomes are affected by age in hypertrophic cardiomyopathy (HCM).

**Methods:**

A cohort of 835 patients with HCM initially evaluated during 2007–2019 were followed for a median of 6.4 years. Study outcomes were all-cause death, cardiovascular and non-cardiovascular death, sudden cardiac death (SCD)/SCD equivalent events, heart failure (HF) events, and the composite cardiovascular outcome including cardiovascular death, SCD/SCD equivalent events, admission for HF, and heart transplantation.

**Results:**

Women were 5 years older (women 59.9±13.5 vs. men 54.9±11.4 years), had worse dyspnea, and greater left ventricular (LV) diastolic dysfunction and obstructive physiology at presentation. Women compared to men had higher all-cause mortality and cardiovascular event rates, driven by more cardiovascular deaths and heart failure (HF) events. Conversely, non-cardiovascular mortality was not different between the sexes. Female sex was independently associated with all-cause death (HR 1.88, 95% CI 1.11–3.20) and composite cardiovascular events (HR 3.60, 95% CI 2.00–6.49), independent of age, body mass index, New York Heart Association class, SCD risk score, and LV ejection fraction. When stratified by the age of 60, sex differences were not significant at <60 years; however, at ≥60 years, women had worse LV diastolic function, greater obstructive physiology, as well as worse survival and composite cardiovascular outcomes. Sex differences in outcomes remained consistent after propensity score matching for age and other clinical characteristics.

**Conclusions:**

Women with HCM have worse cardiovascular prognosis than men, driven by higher cardiovascular mortality and HF events. The negative impact of female sex on cardiac function and cardiovascular outcome became prominent at age ≥60 years, suggesting age-related sex differences in the prognosis of HCM.

## Introduction

Hypertrophic cardiomyopathy (HCM) is the most common inherited cardiomyopathy with an autosomal dominant trait [1]; however, clinical features have been reported to vary according to sex in large cohort studies. Specifically, the prevalence of HCM is higher in men [2, 3]. Also, women are older, have worse symptoms of dyspnea, higher prevalence of obstructive physiology, worse left ventricular (LV) diastolic function, and worse exercise capacity [4–9]. However, data regarding clinical outcomes are conflicting with some studies reporting no sex differences in overall mortality [4, 7], and others reporting that women have worse survival than men in HCM [5, 10]. On the other hand, most studies consistently report a higher risk of heart failure (HF) progression in women [4, 7, 8, 10].

The heart progressively remodels with age. Representative age-related cardiac remodeling involves increasing LV wall thickness in a concentric pattern, and there are sex-specific differences in this process which may affect sex differences in patients with HCM; however, there is a paucity of data. Of note, the risk of adverse outcomes was highest among women aged >50 years in a HCM cohort [4]. Conflicting reports mentioned above regarding sex differences in the clinical outcomes of HCM patients may be related to the different age distributions. Aging-related issues are clinically important, given the extended longevity of patients with HCM caused by recent advances in the contemporary management of HCM [11].

Therefore, in a large multicenter cohort of HCM patients, we aimed to clarify the sex differences in various cardiovascular and non-cardiovascular outcomes, and to investigate whether the sex differences in clinical presentation and outcomes are affected by age.

## Materials and methods

### Study population

This observational cohort included adult HCM patients who underwent index evaluation at two tertiary university centers between January 2007 and May 2019. HCM was diagnosed by increased end-diastolic LV wall thickness (LVWT) ≥15 mm (or ≥13 mm in patients with a family history of HCM) on echocardiography in the absence of other causes of LV hypertrophy [1]. Exclusion criteria were diagnosis of HCM-mimicking diseases such as Fabry disease, glycogen storage disease, cardiac amyloidosis, Noonan syndrome, and mitochondrial disease, or presence of congenital heart disease.

Data on family history of sudden cardiac death (SCD) and history of unexplained syncope were obtained. Patients underwent 24-hour Holter monitoring, and the presence of non-sustained ventricular tachycardia was documented. Medications at index date were reviewed. Patients underwent cardiac magnetic resonance (CMR) imaging at the attending physician’s discretion during the study period, and the presence and extent of late gadolinium enhancement (LGE) were assessed. Details of echocardiographic and CMR examination are available in **Supplementary Methods in S1 File**. The 5-year SCD risk score was calculated according to the European Society of Cardiology guidelines [1]. This study conforms to the principles of the latest declaration of Helsinki in 2013, and the institutional review board of Seoul National University Hospital approved the study protocol (H-2009-013-1154). Written informed consent was waived owing to the retrospective nature of the study.

### Clinical outcomes

The study endpoints were i) all-cause mortality including cardiovascular and non-cardiovascular deaths, ii) SCD/SCD equivalent events including SCD and appropriate implantable cardioverter-defibrillator (ICD) therapy, iii) HF events, and iv) composite cardiovascular outcomes. Cardiovascular death was defined as death caused by cardiac arrest, HF, myocardial infarction, and ischemic or hemorrhagic stroke. Non-cardiovascular death included all other causes of deaths. HF events included progression to New York Heart Association (NYHA) functional class III or IV, first unplanned admission due to HF, heart transplantation due to end-stage HF, and HF-related death. The composite cardiovascular outcome included cardiovascular death, SCD/SCD equivalent events, admission for HF, and heart transplantation. Data on clinical outcomes were collected from the electronic medical records and national death registration database. The index date was designated as the day of the index echocardiographic examination. Patients were followed up until the occurrence of clinical outcomes of interest, the end of the study (August 31, 2019), or censoring due to loss of follow-up.

### Statistical analysis

Continuous data were presented as mean ± standard deviation or median (interquartile range), and categorical data as frequencies and percentages. Patients’ characteristics were compared using Student’s t-test or χ^2^ test, as appropriate. Incidence rates were presented per 100 person-years with 95% confidence interval (CI) estimates based on the Poisson distribution. Kaplan-Meier event-free survival curves for the study endpoints were constructed according to sex and were compared using the log-rank test. Hazard ratios (HR) and 95% CI were calculated using the univariable and multivariable Cox proportional hazard regression models. The proportional-hazards assumption was confirmed using the Schoenfeld residuals. Multivariable Cox regression models were constructed by adjusting for baseline clinical variables that differed significantly between the sexes or were considered clinically important, and by backward stepwise selection from significant variables. The study population was also stratified by the age of 60 years [12], and sex differences in cardiac function and outcomes were explored in each age strata. Considering that the age distribution was the main clinical difference between the sexes, outcomes were compared in the age-matched population. Age was matched in a 1:1 ratio with a 0.2 caliper width using age-based propensity score matching with the nearest neighbor method (R-package “MatchIt”). For sensitivity analysis, outcomes were also compared after propensity score matching between the sexes for clinical variables, specifically, age, body mass index (BMI), family history of HCM, family history of SCD, presence of non-sustained ventricular tachycardia and syncope, advanced symptoms (NYHA class III-IV), the 5-year SCD risk score, prevalence of comorbidities including hypertension, diabetes mellitus, chronic kidney disease, liver disease, ischemic heart disease, atrial fibrillation, and stroke, and use of oral anticoagulants and beta-blockers. A two-sided *p*-value of <0.05 was considered to indicate statistical significance. All statistical analyses were performed with R programming version 4.1.0 (The R foundation for statistical computing, Vienna, Austria).

## Results

### Baseline characteristics

Among the 835 patients, male predominance was noted (n = 612, 73.3%). The baseline characteristics of the study population according to sex are presented in **Table 1**. At the initial evaluation, women were 5 years older, had a lower BMI, and had a higher degree of dyspnea (NYHA class III–IV) than men (age: 59.9 ± 13.5 vs. 54.9 ± 11.4 years; BMI: 24.4 ± 3.6 vs. 25.4 ± 2.8 kg/m^2^; and dyspnea: 8.1% vs. 2.0%; respectively). However, there were no significant differences in the SCD risk factors, SCD risk scores, and the distribution of other comorbidities between the sexes. There was also no significant sex difference in the prescription of oral anticoagulants, beta-blockers, dihydropyridine calcium channel blockers, and angiotensin-converting enzyme (ACE) inhibitors or angiotensin II receptor blockers (ARBs), while non-dihydropyridine calcium channel blockers and diuretics were prescribed more frequently in women.

**Table 1:** Baseline characteristics of the total study population.

	Total (n = 835)	Women (n = 223)	Men (n = 612)	p-value
Age, years	56.3 ± 12,2	59.9 ± 13.5	54.9 ± 11.4	<0.001
Age <60 years	351 (42.0)	89 (39.9)	395 (64.5)	<0.001
Body mass index, kg/m^2^	25.2 ± 3.1	24.4 ± 3.6	25.4 ± 2.8	<0.001
Family history of HCM	77 (9.2)	25 (11.2)	52 (8.5)	0.287
Family history of SCD	107 (12.8)	36 (16.1)	71 (11.6)	0.105
Non-sustained ventricular tachycardia	154 (18.4)	44 (19.7)	110 (18.0)	0.632
Syncope history	116 (13.9)	36 (16.1)	80 (13.1)	0.307
Dyspnea, NYHA class				<0.001
NYHA class I-II	810 (97.0)	205 (91.9)	600 (98.0)	
NYHA class III-IV	25 (3.0)	18 (8.1)	12 (2.0)	
5-year SCD risk score (%)	2.7 ± 2.3	2.6 ± 1.8	2.7 ± 2.5	0.419
5-year SCD risk categories				0.642
Low risk (<4%)	699 (83.7)	190 (85.2)	509 (83.2)	
Intermediate risk (4–6%)	80 (9.6)	21 (9.4)	59 (9.6)	
High risk (≥6%)	56 (6.7)	12 (5.4)	44 (7.2)	
Hypertension	351 (42.0)	99 (44.4)	252 (41.2)	0.503
Diabetes mellitus	142 (17.0)	39 (17.5)	103 (16.8)	0.904
Chronic kidney disease	18 (2.2)	5 (2.2)	13 (2.1)	>0.999
Liver disease	46 (5.5)	8 (3.6)	38 (6.2)	0.194
Ischemic heart disease	106 (12.7)	28 (12.6)	78 (12.7)	>0.999
Atrial fibrillation	110 (13.2)	29 (13.0)	81 (13.2)	>0.999
Stroke	74 (8.9)	17 (7.6)	57 (9.3)	0.533
Baseline medication				
Use of oral anticoagulants	63 (7.5)	15 (6.7)	48 (7.8)	0.695
Use of beta-blockers	286 (34.3)	88 (39.5)	198 (32.4)	0.067
Use of calcium channel blockers (non-dihydropyridine)	113 (13.5)	40 (17.9)	73 (11.9)	0.033
Use of calcium channel blockers (dihydropyridine)	104 (12.5)	27 (12.1)	77 (12.6)	0.948
Use of ACE inhibitors/ARBs	197 (23.6)	59 (26.5)	138 (22.5)	0.278
Use of diuretics	81 (9.7)	35 (15.7)	46 (7.5)	0.001
Echocardiography				
Systolic blood pressure	127 ± 17	125 ± 19	128 ± 16	0.057
Diastolic blood pressure	76 ± 12	72 ± 12	78 ± 11	<0.001
LVEDD, mm	47.6 ± 5.3	45.6 ± 5.2	48.3 ± 5.1	<0.001
Indexed LVEDD, mm/m^2^	27.0 ± 3.6	29.0 ± 4.0	26.2 ± 3.1	<0.001
LVESD, mm	28.2 ± 4.3	27.0 ± 4.3	28.7 ± 4.1	<0.001
Indexed LVESD, mm/m^2^	16.0 ± 2.8	17.2 ± 3.2	15.6 ± 2.5	<0.001
LV ejection fraction (%)	64.5 ± 6.8	64.7 ± 7.5	64.4 ± 6.6	0.649
LV ejection fraction <50%	17 (2.0)	6 (2.7)	11 (1.8)	0.591
Left atrial dimension, mm	44.2 ± 7.1	44.1 ± 7.1	44.3 ± 7.1	0.693
E, m/s	0.63 ± 2.0	0.65 ± 0.21	0.62 ± 0.19	0.070
e’, cm/s	5.2 ± 1.8	4.6 ± 1.9	5.4 ± 1.8	<0.001
s’, cm/s	6.4 ± 1.5	6.1 ± 1.4	6.6 ± 1.6	0.002
E/e’ ratio	13.4 ± 6.1	15.9 ± 7.4	12.5 ± 5.2	<0.001
Pulmonary artery systolic pressure, mmHg	32.3 ± 6.8	33.7 ± 7.8	31.4 ± 6.0	0.013
Max. LV wall thickness, mm	17.0 (15.3–20.0)	17.1 (15.6–20.0)	17.0 (15.2–20.0)	0.414
Indexed max. LV wall thickness, mm/m^2^	9.8 (8.6–11.6)	11.2 (9.9–13.0)	9.4 (8.3–10.8)	<0.001
Max. LVOT gradient ≥30mmHg	123 (14.7)	43 (19.3)	80 (13.1)	0.033
Max. LVOT gradient, mmHg (in obstructive HCM patients)	67 (45–97)	82 (56–116)	60 (40–89)	0.001
LV-GLS (%)	-15.2 ± 4.4	-16.8 ± 4.8	-14.7 ± 4.2	<0.001
Cardiac magnetic resonance available	752 (90.1)	196 (87.9)	556 (90.8)	0.257
Presence of LGE (in patients with CMR)	643 (85.5)	152 (77.6)	491 (88.3)	<0.001
Extensive LGE (in patients with CMR)	199 (26.5)	38 (19.4)	161 (29.0)	0.012

ACE, angiotensin-converting enzyme; ARB, angiotensin II receptor blocker; E, peak early diastolic mitral inflow velocity; e’, early diastolic mitral annular velocity; HCM, hypertrophic cardiomyopathy; ICD, implantable cardioverter defibrillator; LV, left ventricular; LVEDD, LV end-diastolic dimension; LVESD, LV end-systolic dimension; LV-GLS, LV global longitudinal strain; LVOT, LV outflow tract; NYHA, New York Heart Association; SCD, sudden cardiac death; s’, systolic mitral annular velocity.

The index echocardiographic evaluation showed that women had smaller absolute but greater indexed LV dimensions, smaller e’ and s’ velocities, greater E/e’ ratios, greater estimated pulmonary artery systolic pressure (PASP), and a higher proportion of obstructive physiology with greater LV outflow tract (LVOT) gradients than men. Although there was no significant difference in the absolute maximum LV wall thickness between the sexes, the indexed maximum LV wall thickness was significantly higher in women. LV ejection fraction (LVEF) was not different between the sexes, but women had better LV global longitudinal strain (LV-GLS) than men. CMR was available in 90.1% of the patients. The presence of any LGE and extensive LGE was both higher in men compared to women.

### Survival analysis

The median follow-up was 6.4 (4.1–9.2) years. There was no significant difference in the follow-up duration between the sexes (women, 6.1 [3.7–9.0] vs. men, 6.5 [4.2–9.2] years). During follow-up, 64 patients died (7.7%): 32 cardiovascular causes (3.8%) and 32 non-cardiovascular causes (3.8%) (**S1 Table in S1 File**). SCD/SCD equivalent events occurred in 22 patients (2.6%), HF events in 58 patients (6.9%), and the composite cardiovascular outcome in 54 patients (6.5%).

**Fig 1:**
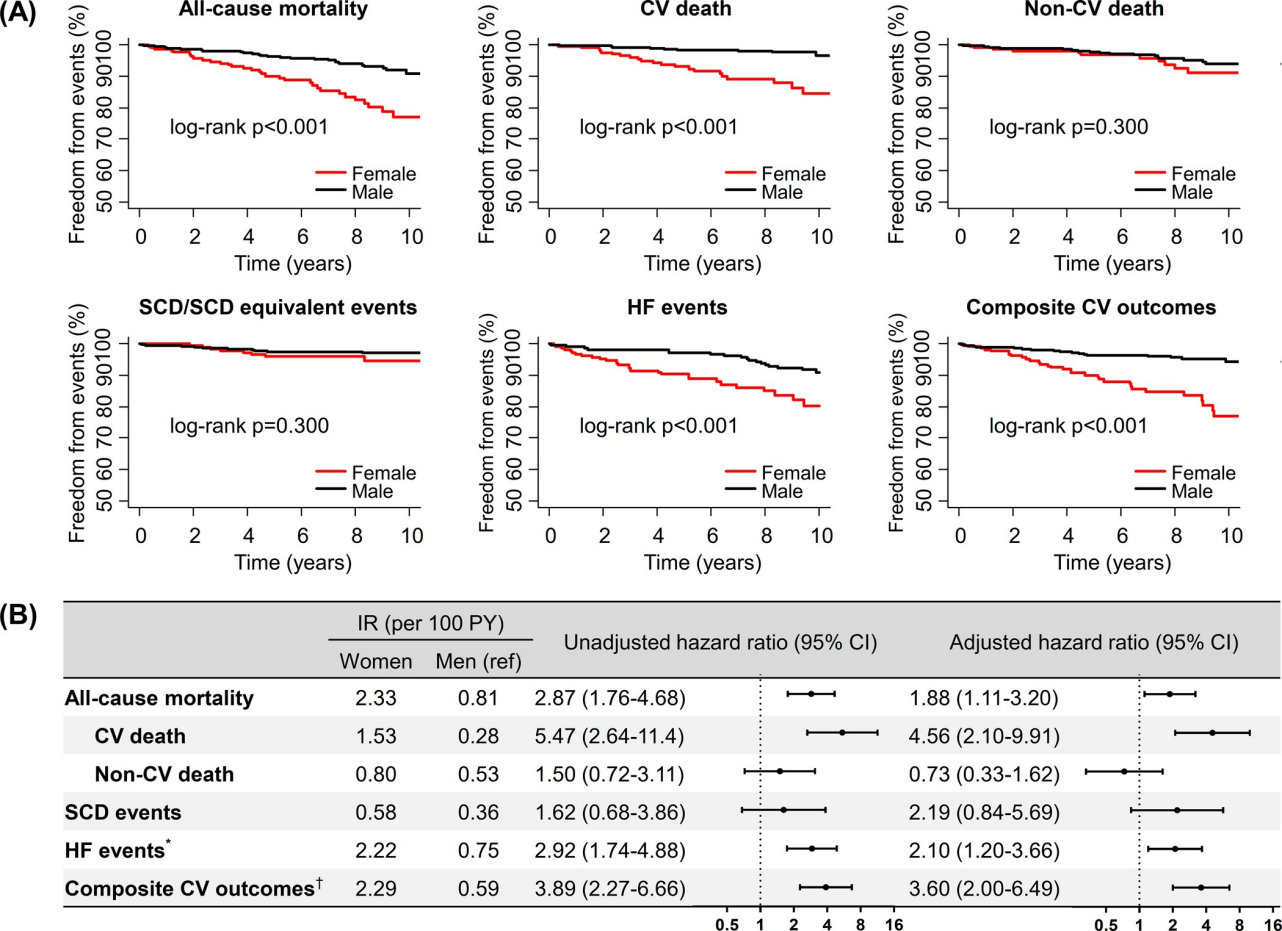
Sex differences in clinical outcomes. (A) Sex differences of event-free survival curves for clinical outcomes. (B) Independent association of sex with various outcomes. Multivariable adjustment was performed for age, body mass index, NYHA class, the SCD risk score, and left ventricular ejection fraction. *HF events = Admission for HF + Heart transplantation + Death due to HF + Progression to NYHA functional class III/IV. ^†^Composite CV outcomes = CV death + SCD/SCD equivalent events + Admission for HF + Heart transplantation. CI, confidence interval; CV, cardiovascular; HF, heart failure; IR, incidence rate; NYHA, New York Heart Association; PY, person-years; SCD, sudden cardiac death.

Kaplan-Meier survival curves (**Fig 1A**) demonstrated that women had a significantly higher all-cause mortality than men, which was primarily driven by higher cardiovascular mortality; however, no significant difference was observed in non-cardiovascular mortality. Women had significantly more HF events than men, while the difference in SCD/SCD equivalent events was not significant. The incidence of composite cardiovascular outcomes was significantly higher in women than in men and was mainly driven by the higher rates of cardiovascular deaths and HF events.

On univariable Cox regression analysis, significant predictors of composite cardiovascular outcomes were female sex, age, NYHA class, atrial fibrillation, stroke, use of oral anticoagulants/diuretics, SCD risk score, and LVEF. Also, significant clinical predictors of all-cause mortality included female sex, age, BMI, NYHA class, chronic kidney disease, liver disease, atrial fibrillation, stroke, use of oral anticoagulants/ACE inhibitors or ARBs/diuretics, and LVEF (**S2 Table in S1 File**).

Multivariable Cox regression analyses adjusting for the clinical differences between the sexes (i.e. age, BMI, and NYHA class), the SCD risk score, and LVEF demonstrated that female sex was independently associated with poor cardiovascular prognosis (**Fig 1B** and **S3 Table in S1 File**). Specifically, female sex was independently associated with higher all-cause mortality (HR 1.88, 95% CI 1.11–3.20) and cardiovascular death (HR 4.56, 95% CI 2.10–9.91), but not with non-cardiovascular death (HR 0.73, 95% CI 0.33–1.62). Female sex was also significantly associated with HF events (HR 4.52, 95% CI 1.74–11.8), but not with SCD/SCD equivalent events (HR 2.19, 95% CI 0.84–5.69). Finally, female sex was independently associated with a higher risk of the composite cardiovascular outcome (HR 3.60, 95% CI 2.00–6.49).

For sensitivity analysis, we also performed multivariable analysis further adjusting for the presence of extensive LGE, in the subset of patients with CMR (**S4 Table in S1 File**). Female sex remained significantly associated with all-cause mortality, cardiovascular death, HF events, and the composite cardiovascular outcome.

### Stratification by age

To analyze the effect of age on prognosis in both sexes, we divided both groups into two, with a cutoff age of 60. There were 89 women and 395 men younger than 60 years old, and 134 women and 217 men aged 60 years or more.

Women showed smaller absolute but larger indexed LV dimensions, higher indexed maximum LV wall thickness, and better LV-GLS than men regardless of age strata (**Table 2**). Compared to patients aged <60 years, the sex difference in indexed maximum LV wall thickness increased while that in LV-GLS decreased in those aged ≥60 years. Furthermore, at ≥60 years old, women showed significantly worse LV diastolic function, greater PASP, and more obstructive physiology compared to men. Meanwhile, at <60 years of age, women had a slightly higher E/e’ ratio (13.9 ± 6.0 vs. 12.4 ± 5.3); however, no other significant difference was observed in the Doppler parameters of LV diastolic function or obstructive physiology compared to men.

**Table 2:** Comparison of echocardiographic characteristics between the sexes at age under and over 60.

	Age <60			Age ≥60		
	Women (n = 89)	Men (n = 395)	p-value	Women (n = 134)	Men (n = 217)	p-value
Systolic blood pressure	119 ± 17	127 ± 16	<0.001	129 ± 19	130 ± 16	0.771
Diastolic blood pressure	70 ± 12	78 ± 12	<0.001	74 ± 11	77 ± 11	0.006
LVEDD, mm	45.1 ± 4.3	47.9 ± 5.3	<0.001	45.9 ± 5.7	49.0 ± 4.7	<0.001
Indexed LVEDD, mm/m^2^	27.9 ± 3.5	25.7 ± 3.1	<0.001	29.8 ± 4.1	27.3 ± 3.0	<0.001
LVESD, mm	26.6 ± 3.8	28.4 ± 4.1	<0.001	27.3 ± 4.6	29.2 ± 4.2	<0.001
Indexed LVESD, mm/m^2^	16.4 ± 3.0	15.2 ± 2.4	0.001	17.7 ± 3.2	16.2 ± 2.5	<0.001
LV ejection fraction (%)	65.2 ± 7.6	64.5 ± 6.7	0.377	64.3 ± 7.4	64.3 ± 6.5	0.990
LV ejection fraction <50%	3 (3.4%)	8 (2.0%)	0.707	3 (2.2%)	3 (1.4%)	0.859
Left atrial dimension, mm	42.3 ± 7.0	43.7 ± 6.7	0.078	45.3 ± 6.9	45.4 ± 7.6	0.856
E, m/s	0.65 ± 0.23	0.63 ± 0.20	0.347	0.65 ± 0.20	0.61 ± 0.18	0.046
e’, cm/s	5.3 ± 2.1	5.5 ± 1.8	0.246	4.2 ± 1.6	5.2 ± 1.7	<0.001
s’, cm/s	6.5 ± 1.3	6.7 ± 1.5	0.379	5.8 ± 1.4	6.4 ± 1.6	0.012
E/e’ ratio	13.9 ± 6.0	12.4 ± 5.3	0.020	17.3 ± 8.0	12.5 ± 5.1	<0.001
Pulmonary artery systolic pressure, mmHg	32.0 ± 6.2	30.9 ± 5.5	0.369	34.7 ± 8.4	32.0 ± 6.2	0.040
Max. LV wall thickness, mm	17.0 (15.5–20.4)	17.1 (15.1–20.0)	0.947	17.8 (15.6–20.0)	17.0 (15.3–18.9)	0.105
Indexed max. LV wall thickness, mm/m^2^	10.5 (9.6–12.8)	9.4 (8.2–11.1)	<0.001	11.3 (10.3–13.2)	9.4 (8.5–10.5)	<0.001
Max. LVOT gradient ≥30mmHg	10 (11.2%)	57 (14.4%)	0.536	33 (24.6%)	23 (10.6%)	0.001
Max. LVOT gradient, mmHg (in obstructive HCM patients)	69 (47–101)	54 (40–76)	0.184	96 (57–118)	80 (47–98)	0.139
LV-GLS (%)	-17.3 ± 4.6	-14.6 ± 4.1	<0.001	-16.4 ± 4.9	-14.7 ± 4.3	0.001

E, peak early diastolic mitral inflow velocity; e’, early diastolic mitral annular velocity; LV, left ventricular; LVEDD, LV end-diastolic dimension; LVESD, LV end-systolic dimension; LV-GLS, LV global longitudinal strain; LVOT, LV outflow tract; s’, systolic mitral annular velocity.

**Fig 2:**
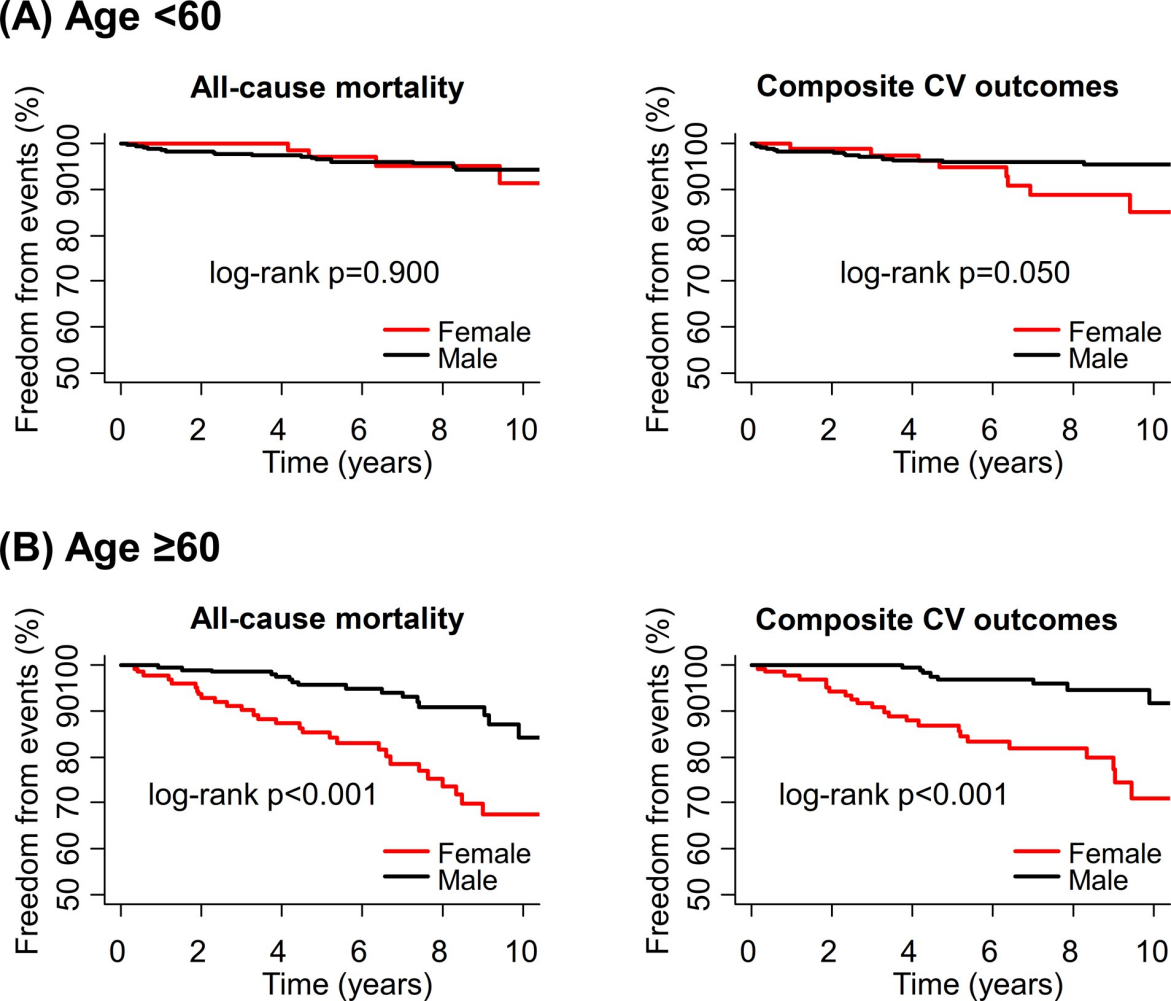
Sex differences in clinical outcomes, stratified by age. (A) Below the age of 60, there was no significant difference in survival and composite cardiovascular outcome between the sexes under the age of 60, though there was a non-significant trend for higher incidence of the latter in women. (B) Over the age of 60, women showed worse survival and higher incidence of the composite cardiovascular outcomes compared to men.

Number of events and incidence rates for clinical outcomes according to sex and age strata are shown in **S5 Table in S1 File**. HCM patients aged ≥60 years had higher all-cause mortality and composite cardiovascular outcomes compared to HCM patients aged <60 years (**S1 Fig in S1 File**). When divided by the sex, outcomes in elderly HCM women were significantly worse compared to young HCM women, but there was no significant difference in outcomes between elderly and young HCM men. Consequently, sex differences in outcomes emerged more clearly at advanced age. Kaplan-Meier survival analyses demonstrated that women had markedly worse survival and cardiovascular outcome-free survival compared to men at age ≥60 years; however, these differences in survival were not significant below the age of 60 (**Fig 2**). Thus, the subgroup of women aged ≥60 years had the worst survival and cardiovascular outcome-free survival, compared with the other subgroups of men aged ≥60 years or women aged <60 years (**Fig 2** and **S1 Fig in S1 File**).

### Sex differences after age-matching and propensity score matching

In the total HCM cohort, the age distribution was the main clinical difference between men and women, with women being older (**Table 1**). To balance the age difference between the sexes, we performed 1:1 matching of women and men using age-based propensity scores, and 218 pairs of women and men were finally matched. The distribution of age was well balanced between the sexes in the age-matched HCM cohort (**S2 Fig in S1 File**). Other baseline clinical characteristics did not differ between the age-matched women and men, with the only exception being a higher degree of dyspnea in women (**S6 Table in S1 File**). Similar to the echocardiographic features observed in the total cohort, women had worse diastolic function evidenced by lower e’ velocities and greater E/e’ ratio, as well as higher LVOT pressure gradients, greater indexed maximum LV wall thickness, and better LV-GLS, while there was no significant difference in LVEF and absolute maximum LV wall thickness.

The number of events for each endpoint in the age-matched cohort are presented in **S7 Table in S1 File**. The survival analysis for each endpoint remained the same as those obtained from the original HCM cohort (**S3 Fig in S1 File**). Specifically, women had a significantly higher all-cause mortality with higher cardiovascular mortality, but no difference in non-cardiovascular mortality. In addition, women had a significantly higher incidence of HF events. Women also had a higher incidence of the composite cardiovascular outcome than the age-matched men.

Survival analyses stratified by the age of 60 years in the age-matched cohort also showed results consistent with those observed in the original HCM cohort (**S4 Fig in S1 File**). Women showed significantly higher mortality or composite cardiovascular outcomes compared to men over the age of 60, while there was no significant difference in outcomes between the sexes under the age of 60.

As a sensitivity analysis, we also performed propensity score matching of women and men using propensity scores constructed with age and all other clinical variables, and 209 pairs were finally matched (**S5 Fig in S1 File**). Clinical characteristics were well balanced between the sexes after propensity score matching (**S8 Table in S1 File**). Sex differences in echocardiographic features were consistent after propensity score matching (**S9 Table in S1 File**). Survival analyses for the endpoints in the total matched cohort and after stratification by age also showed consistent results with the original HCM cohort (**S10 Table, S6 and S7 Figs in S1 File**).

## Discussion

In a large cohort of patients with HCM, we demonstrated the presence or absence of sex differences and its relation to age in various cardiovascular and non-cardiovascular outcomes. Specifically, women had higher cardiovascular mortality and more HF events than men with no difference seen in non-cardiovascular mortality. Importantly, we demonstrated that aging may affect the sex differences in HCM. When stratified by the age of 60 years, the negative impact of female sex on cardiac function and cardiovascular outcome became clear at ≥60 years of age. While sex differences were not significant below the age of 60, women had greater LV diastolic dysfunction, obstructive physiology, as well as worse survival and cardiovascular outcomes than men at ≥60 years. All of these findings were consistently observed in the age-matched and propensity score-matched cohort.

### Sex differences and age-dependency in clinical outcomes in HCM

Male predominance of approximately 60–70% and older age of women have been consistently observed in previous large HCM cohorts [2–8, 13, 14], and in our study. Women with HCM have greater obstructive physiology and LV diastolic dysfunction, which are predictors of HF progression and death in HCM patients [15, 16]. However, there is some discrepancy in previous reports of sex differences in the clinical outcomes of HCM patients. While higher risk of HF is consistently reported in women [4, 7, 8, 10, 13, 14], there are conflicting results concerning sex differences in all-cause mortality in HCM: some studies have shown increased all-cause mortality in women [5, 10, 13], while others have reported no sex differences in mortality [4, 7].

We observed that women with HCM had higher all-cause mortality and composite cardiovascular events, driven by higher cardiovascular mortality and more HF events, while no differences were seen in non-cardiovascular mortality and SCD/SCD equivalent events. This was consistent after age-matching and propensity-score matching. Interestingly, we observed that the effect of sex on clinical outcomes seemed to vary with age. Below the age of 60 years, there was no difference in the all-cause mortality and composite cardiovascular outcomes between the sexes. However, over the age of 60 years, women had significantly higher all-cause mortality and composite cardiovascular outcomes than men, and the difference between the sexes became more apparent. Elderly women with HCM had the worst prognosis. This is of greater clinical importance in the contemporary era, considering the extended life expectancy of HCM subjects [11] and increasing diagnosis of HCM in elderly subjects [17, 18].

Sex- and age-related adverse outcomes in older women may be explained by the greater worsening of LV diastolic function and obstructive physiology with aging compared to men. Regardless of age, women with HCM had smaller LV cavities with similar degree of maximum LV wall thickness (but greater indexed maximum LV wall thickness) compared with men. However, worse LV diastolic function, and obstructive physiology in women became prominent after ≥60 years. Women also had greater estimated PASP than men; pulmonary hypertension was reported to be more common in women with HCM, and was associated with worse outcomes [19, 20].

The discrepancy of reported sex differences in clinical outcomes may be explained by the different age distributions of the HCM cohorts, and the sex differences in mortality may have manifested more clearly in the older cohorts. A study including an older HCM population (mean age, 55 years; similar to ours) found worse survival in women than in men [5], while two large studies with relatively younger HCM patients (mean age, 42 and 46 years, respectively) did not find any difference between the sexes [4, 7]. Also, in one of the latter studies, the risk of HF-related clinical deterioration and death was reported to be greater in women aged ≥50 years than in women aged <50 years or in men with HCM [4]. A recent nationwide study including only HCM patients aged ≤65 years found higher rates of HF admission in women but no difference in cardiovascular death or all-cause death [14].

### Sex-specific patterns of cardiac aging as a mechanism for age-related sex differences in HCM

The normal cardiac aging process may contribute to the accentuation of sex differences in HCM at older age. Different patterns of cardiac remodeling between the sexes with advancing age are reported in the general population; women experienced a steeper increase in LV wall thickness, more concentric LV remodeling, and greater worsening of LV diastolic function than men [21–23], thus, predisposing older women to HF with preserved ejection fraction [24]. This tendency for concentric remodeling in women is likely to contribute to greater obstructive physiology in older women with HCM. The postmenopausal decline of the estrogen levels is related to the changes in the renin-angiotensin-aldosterone system, natriuretic peptide levels, and increased oxidative stress and extracellular matrix in the myocardium, which can contribute to aggravating HF [25]. Interestingly, postmenopausal estrogen replacement was reported to be associated with decreased LV mass and improved LV diastolic function in the general population, which further supports the role of estrogen in cardiac remodeling [26, 27]. Age-related changes, especially postmenopausal estrogen withdrawal, are likely to affect the clinical course and outcomes in women with HCM, which is a future topic of research interest in HCM.

Interestingly, LV-GLS was better preserved in women than men, though the difference was reduced at an older age (≥60 years). In the general population, women were also reported to have higher absolute LV-GLS compared with men [28], although it is known that women are more predisposed to HF with preserved ejection fraction [24]. This suggests that a mismatch in systolic and diastolic function is more evident in women, with and without HCM, and this may also contribute to reducing the mechanical efficiency of the heart and subsequently predisposing to HF development.

### Biological evidence of sex differences in HCM

Animal models of HCM provide biological evidence that female sex is related to lower penetrance of the genetic mutation, delayed clinical expression of LV hypertrophy, and LV diastolic dysfunction that is aggravated with estrogen withdrawal, all of which are in line with the observations in our study. Female sex was initially protective against disease manifestation and progression in a transgenic murine HCM model with myosin heavy-chain mutation, suggesting that sex-related factors play a role in the expression of sarcomeric gene mutations [29, 30]. Even with the same genetic mutation, sex-related differences in myofilament function and growth were reported [31]. These findings may explain the lower penetrance or delayed expression of HCM in women and the male predominance. A murine model with cardiac troponin C mutation demonstrated that female mice had worse LV diastolic function than male mice and that there were sex differences in the cardiac transcriptomes [32]. Moreover, female mice with troponin T mutation showed advanced LV diastolic dysfunction and increased myocardial oxidative stress with ovariectomy, which subsequently improved with estrogen replacement [33]. This supports the negative effect of postmenopausal hormonal changes on elderly HCM women.

### Other factors to consider in sex differences

Other factors may contribute to sex differences in HCM. A previous study suggested that delayed diagnosis in women might explain the sex differences observed in HCM, based on the observation that women with HCM were older than men and were less commonly diagnosed incidentally [4]. Underrepresentation of women with HCM may be related to the lack of awareness for cardiovascular risk [34], less exposure to medical screening programs [34], and vague symptoms of dyspnea that are similar to those observed in women with HF and preserved ejection fraction. However, in our study, sex differences in the cardiac function and clinical outcomes remained significant in the propensity score-matched cohort, suggesting that these clinical differences were not solely attributable to the delayed diagnosis in women. Other disparities in the socio-economic and psychological factors can contribute to sex differences and must not be overlooked [35].

Considering that the diagnostic criteria for HCM in terms of LV wall thickness do not differ between women and men despite the inherent differences in total cardiac mass [1], women may be in a more advanced stage of the disease to reach the diagnostic threshold for HCM [36, 37], which in turn can lead to worse clinical outcomes. We observed that the absolute maximum LV wall thickness was not different between the sexes; however, when indexed to body surface area, it was greater in women than in men, supporting the possibility of more advanced disease in women with HCM.

### Limitations

First, there may be referral bias, as the study population was recruited from two tertiary referral centers. However, a large proportion of asymptomatic or less symptomatic patients diagnosed by routine health examinations were included, as evidenced by a high proportion of patients with NYHA class I or II dyspnea. Second, the outcomes were retrospectively analyzed, and there are limitations inherent to a retrospective study design. Third, we performed multivariable adjustments and propensity score matching to control for clinical differences between the sexes, but there may be residual confounding we did not consider. Moreover, including all variables for multivariable adjustment was infeasible. Study findings should be validated in a separate cohort. Fourth, this was an Asian population, and the possibility must be considered that race and ethnicity may contribute to sex differences shown in HCM and its underlying molecular mechanisms. Finally, we did not directly track the longitudinal changes in the cardiac structure and function in patients with HCM, especially in women before and after menopause. This issue is worthy of further investigation.

## Conclusions

Overall, women with HCM had worse cardiovascular outcomes than men, predominantly driven by more cardiovascular deaths and HF events. Non-cardiovascular mortality was not different between the sexes. Importantly, the negative impact of female sex on cardiac function and cardiovascular outcome became prominent at ≥60 years old, while sex differences were not significant below the age of 60. Based on these findings, clinicians should more carefully monitor elderly women with HCM to improve prognosis.

## Supporting information

S1 File (DOCX)
