# Supplementary material for: Age-related sex differences in the outcomes of patients with hypertrophic cardiomyopathy
Source: PLoS One. 2022 Feb 25;17(2):e0264580. doi: 10.1371/journal.pone.0264580 (PMC8880392; doi:10.1371/journal.pone.0264580)
Supplement: S1 File — (DOCX) [file pone.0264580.s001.docx]

**Supplemental Materials**

**Age-related sex differences in the outcomes of
patients with hypertrophic cardiomyopathy**

Hyun-Jung Lee, MD^1^, Steve R. Ommen, MD^2^, ^*^Hyung-Kwan Kim, MD, PhD^1^, ^*^Sang Chol Lee, MD, PhD^3^, Jihoon Kim, MD^3^, Jun-Bean Park, MD, PhD^1^, You-Jung Choi, MD^1^,
Seung-Pyo Lee, MD, PhD^1^, Sung-A Chang, MD, PhD^3^, Yong-Jin Kim, MD, PhD^1^

^1^Cardiovascular Center, Seoul National University Hospital, Seoul National University School of Medicine, Seoul, Korea

^2^Division of Cardiovascular Diseases, Mayo Clinic College of Medicine, Rochester, Minnesota, USA

^3^Cardiovascular Imaging Center, Heart Vascular Stroke Institute, Samsung Medical Center, Sungkyunkwan University School of Medicine, Seoul, Korea

**^*^**These two authors equally contributed to this work as corresponding authors.

**Table of Contents**

1. **Supplementary Methods**
2. **Supplementary Figures**
3. **Supplementary Tables**
4. **Supplementary References**

**­**

**Supplementary Methods**

*Echocardiographic examination*

Transthoracic echocardiography was performed according to the guidelines,[1] including measurements of LV end-systolic/end-diastolic dimensions, LV end-diastolic interventricular septal/posterior wall thickness, and LV ejection fraction (LV-EF). LA dimension was measured from the parasternal long-axis view. The maximum LV end-diastolic wall thickness was measured. The LV dimensions and maximum LV wall thickness were also indexed to body surface area. From the mitral inflow Doppler signals, early transmitral inflow velocity (E) was obtained with the sample volume placed between the tips of mitral leaflets. Pulsed-wave tissue Doppler imaging technique was used to measure peak systolic (s') and early diastolic (e') mitral annular velocity at the septal mitral annulus in the apical 4-chamber view. Pulmonary artery systolic pressure was derived by addition of estimated right atrial pressure and trans-tricuspid gradient calculated from the modiﬁed Bernoulli equation [4*(peak tricuspid regurgitation velocity)^2^] in the absence of pulmonary stenosis or right ventricular outﬂow tract obstruction. Right atrial pressure (5, 10, 15-20 mmHg) was based on inferior vena cava size and collapsibility. LV outflow tract (LVOT) pressure gradient was measured with continuous-wave Doppler from the apical window at rest and during Valsalva maneuver, and the maximum value was recorded. Obstructive physiology was considered to be present if maximum LVOT gradient was ≥ 30 mmHg at rest or during Valsalva maneuver. LV global longitudinal strain (LV-GLS) was calculated as the average of segmental longitudinal strain values obtained from all three apical views, using an offline vendor-independent post-processing software (TomTec Imaging Systems, version 4.6, Munich, Germany).

*Cardiac magnetic resonance imaging*

Patients underwent cardiac magnetic resonance (CMR) imaging during follow-up at the attending physician’s discretion. CMR was performed with available equipment, specifically, a 1.5-T (Achieva, Philips Medical Systems, Best, Netherlands; Samsung Medical Center) or 1.5-T/3-T (Magnetom Trio, Siemens, Erlangen, Germany; Seoul National University Hospital) scanner, as described in a previous study.[2, 3] Images for late gadolinium enhancement (LGE) analysis were acquired 10 to 20 minutes after contrast (0.2mmol/kg gadopentetate or 0.1mmol/kg gadobutrol) administration. The presence and extent of LGE was assessed visually on short-axis images in phase sensitive inversion recovery sequence. The presence of any LGE, including right ventricular insertion sites to the ventricular septum were assessed. Extensive LGE was defined as being extensive and diffuse by visual inspection, or comprising ≥15% of LV mass, according to the guidelines.[4]

**Supplementary Figures**


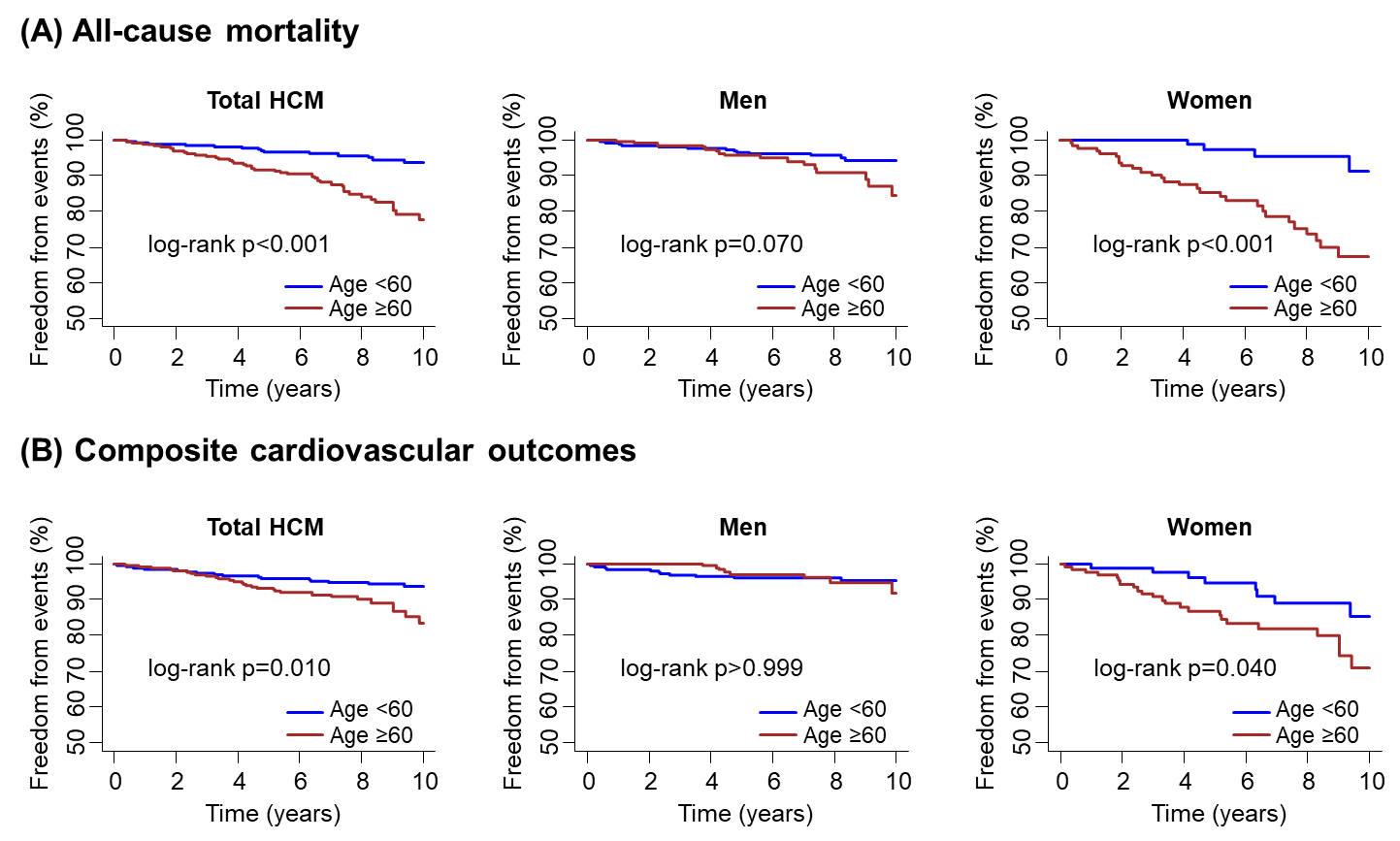


**S1 Fig. Clinical outcomes according to age, stratified by sex.**

HCM patients aged ≥60 years had higher all-cause mortality and composite cardiovascular outcomes compared to HCM patients aged <60 years. When stratified by the sex, all-cause mortality and composite cardiovascular outcomes were significantly higher in elderly HCM women compared to young HCM women; meanwhile, there was no significant difference in outcomes between elderly and young HCM men.

**
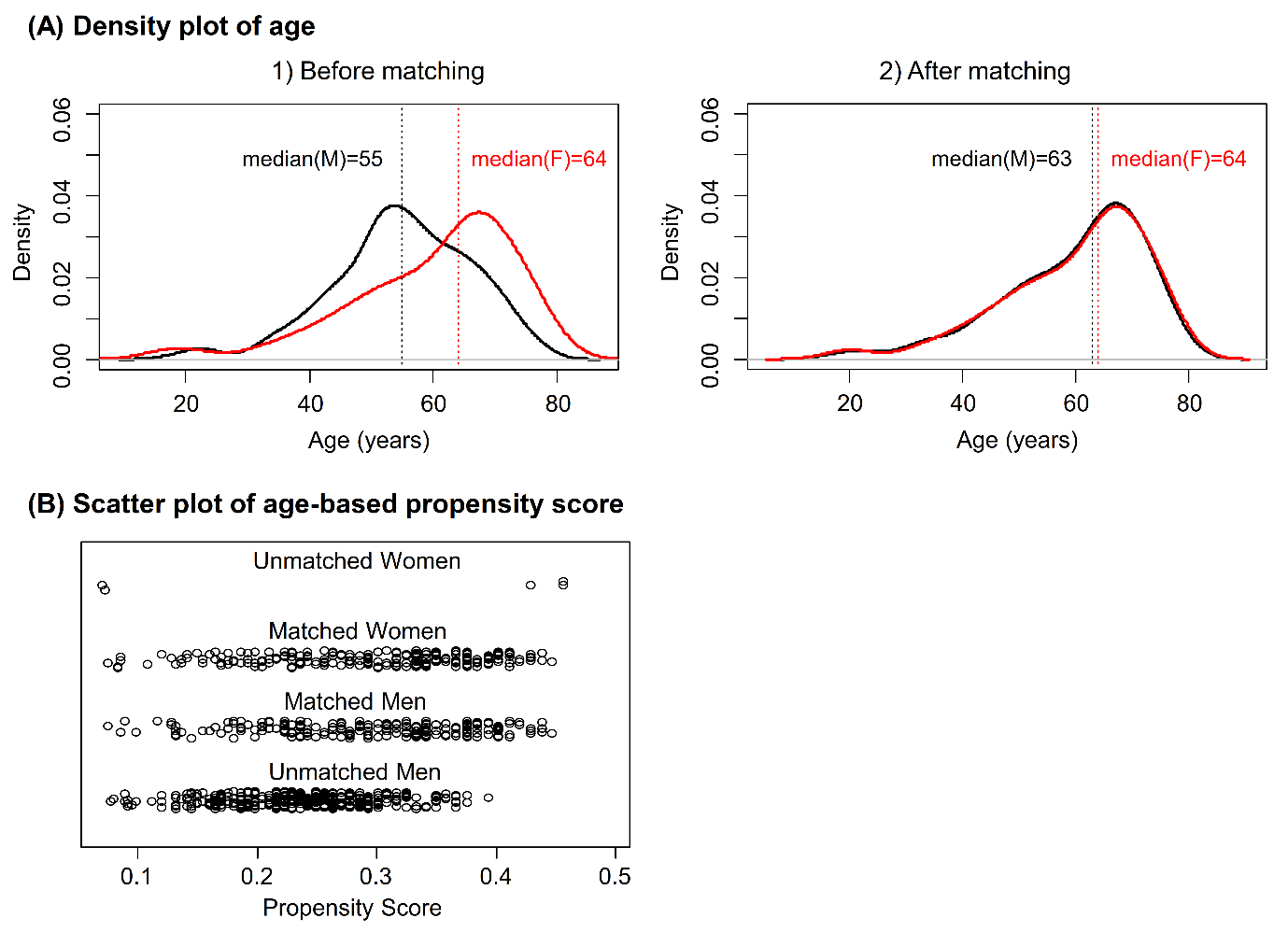
**

**S2 Fig. Balance of age between the sexes before and after age-matching**

(A) Age distributions were different between the sex in the original study population, and we performed 1:1 matching of the sexes using age-based propensity scores and the nearest neighbor method with a 0.2 caliper width. As a result, 218 women and 218 men were finally matched, and 5 women were excluded. (B) After matching, age was well balanced between the sexes.

**
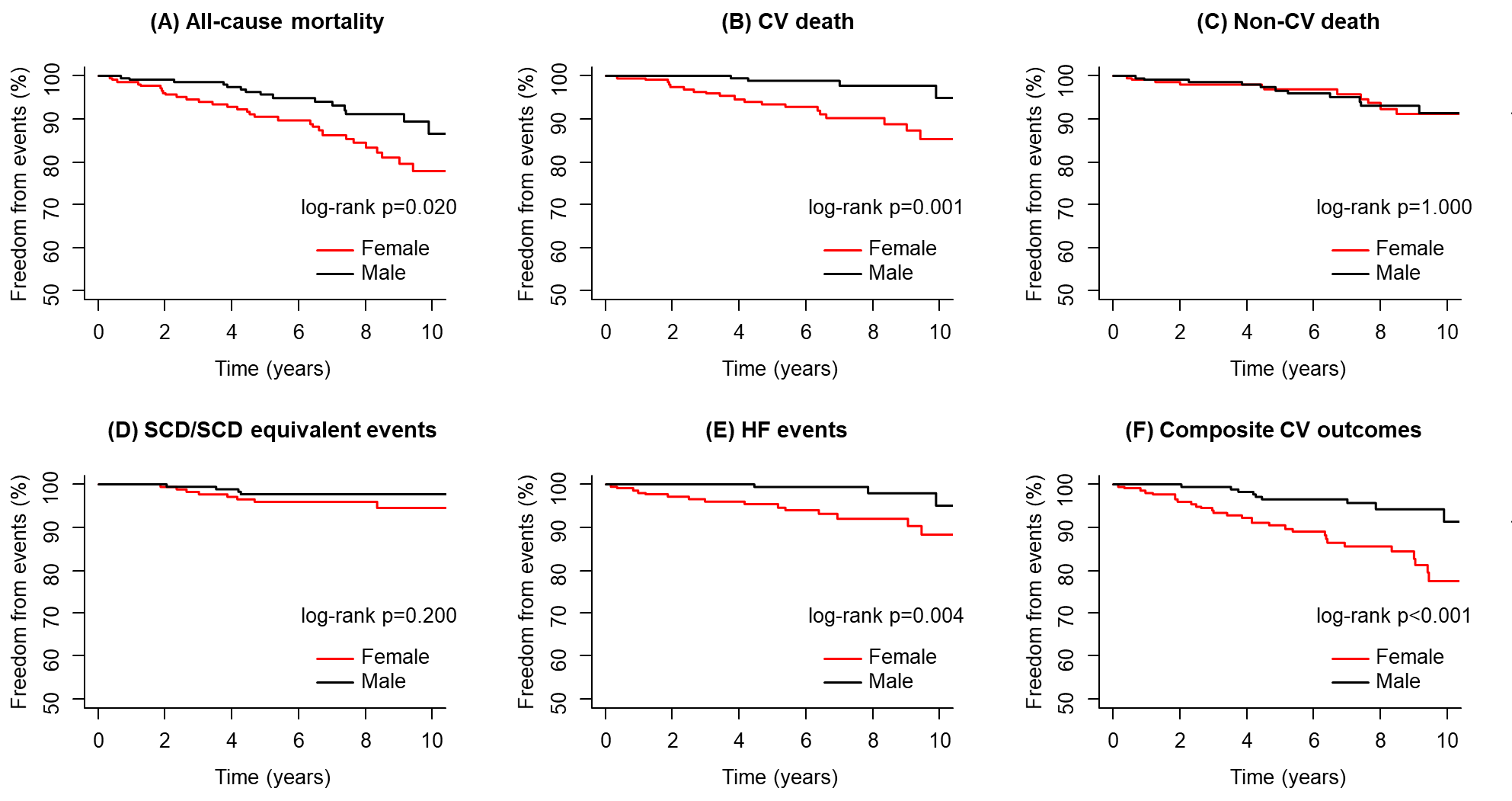
**

**S3 Fig. Sex differences of event-free survival curves for clinical outcomes, in the age-matched cohort of women and men**

Survival analyses for each endpoint remained consistent with those obtained from the original hypertrophic cardiomyopathy cohort. In brief, women showed significantly a higher all-cause mortality, CV death, HF events, and composite CV outcomes. Meanwhile, there was no significant difference in non-CV death or SCD events between the sexes.

CV, cardiovascular; HF, heart failure; SCD, sudden cardiac death.

**
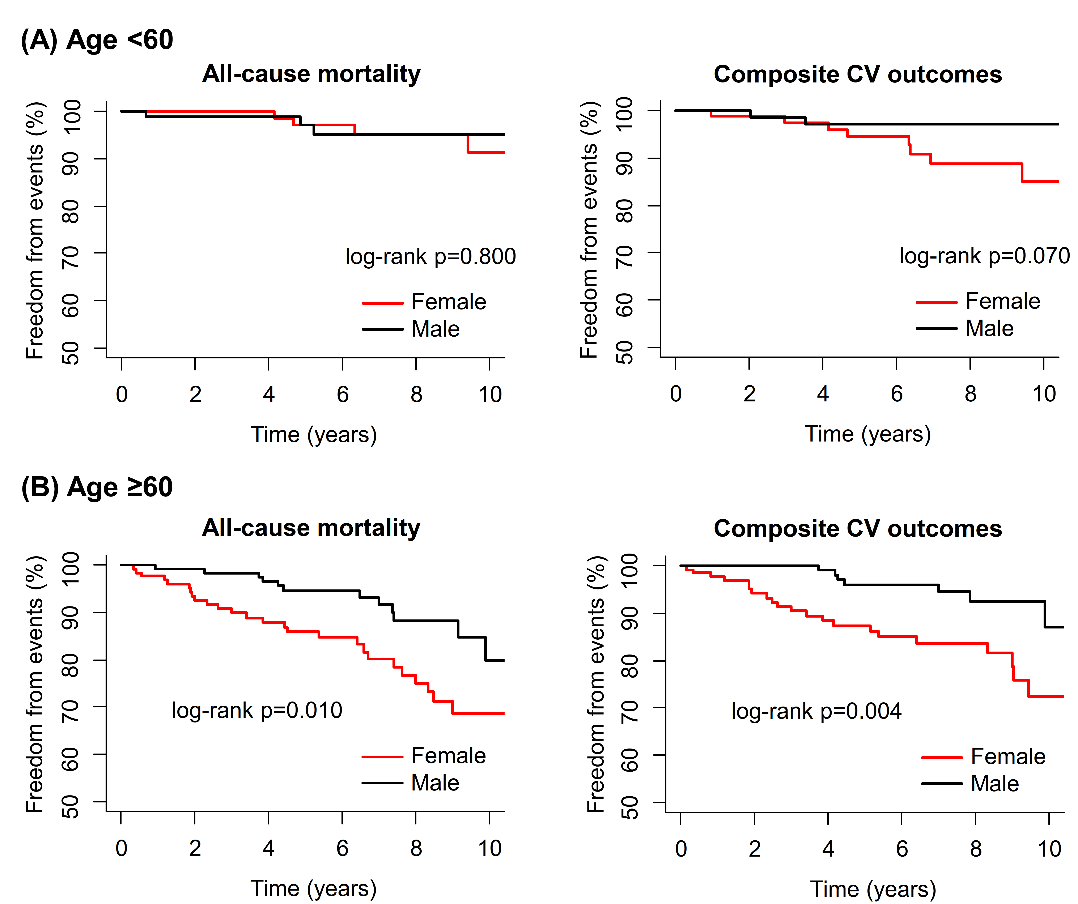
**

**S4 Fig. Sex differences of event-free survival curves for all-cause mortality and composite cardiovascular outcomes, stratified by age of 60 years, in the age-matched cohort**

Results were consistent with those of the original HCM cohort.

**
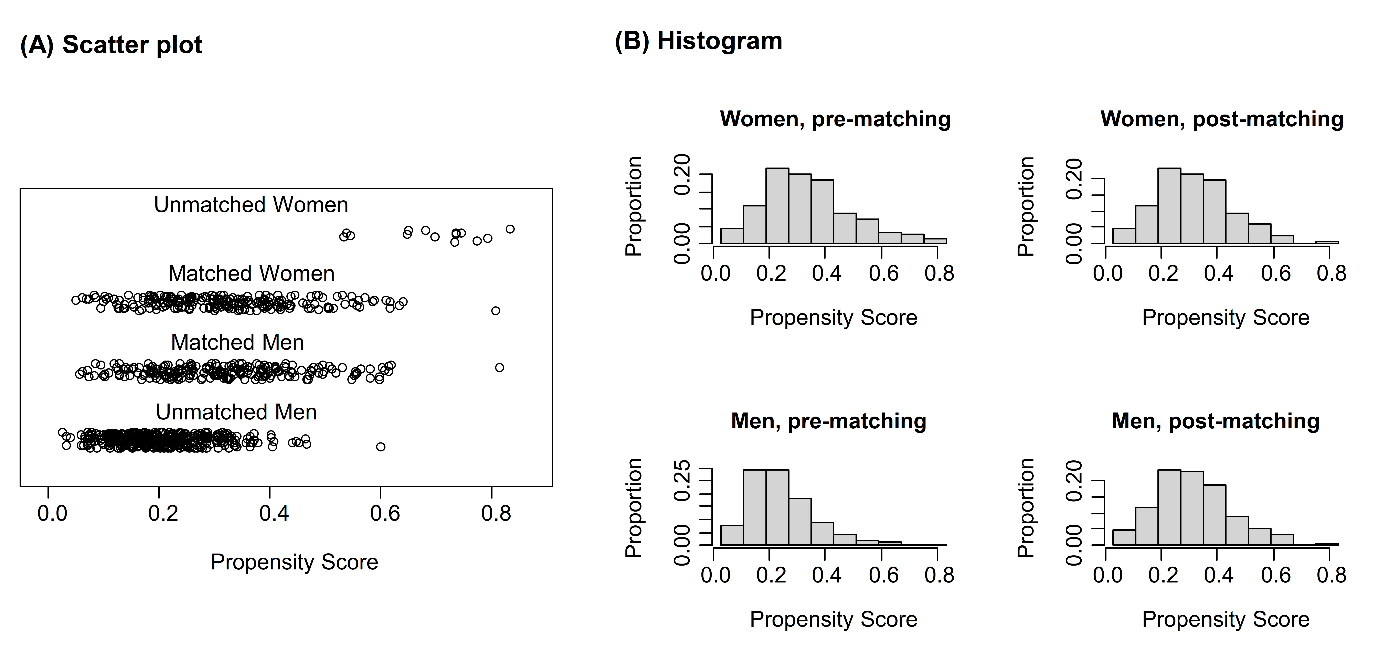
**

**S5 Fig. Distribution of propensity scores before and after propensity score matching**

After matching, propensity scores were well balanced between the sexes.


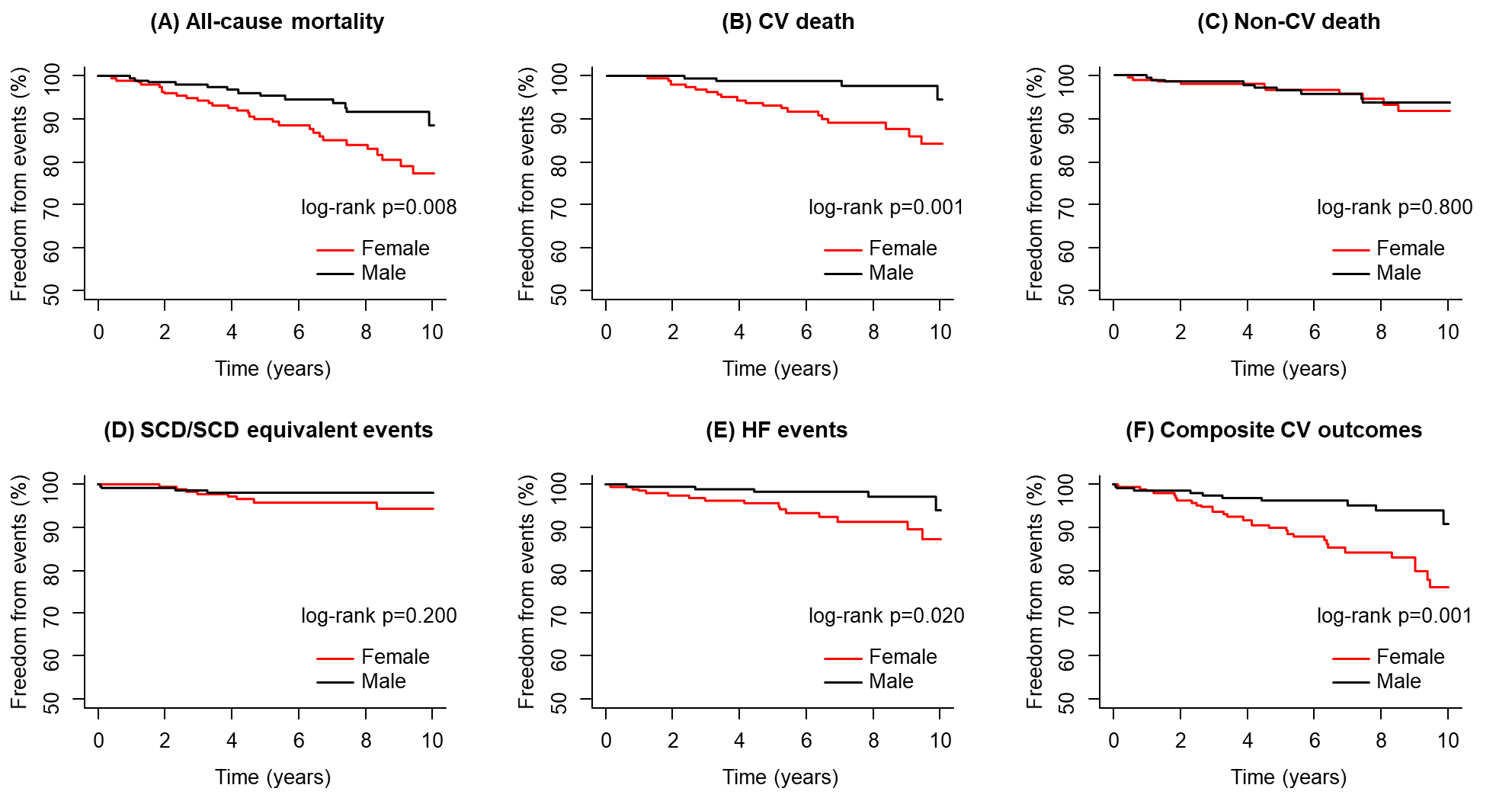


**S6 Fig. Sex differences of event-free survival curves for clinical outcomes, after propensity score matching**

Survival analyses for each endpoint remained consistent with those obtained from the original hypertrophic cardiomyopathy cohort. In brief, women showed significantly a higher all-cause mortality, CV death, HF events, and composite CV outcomes. Meanwhile, there was no significant difference in non-CV death or SCD events between the sexes.

CV, cardiovascular; HF, heart failure; SCD, sudden cardiac death.

**
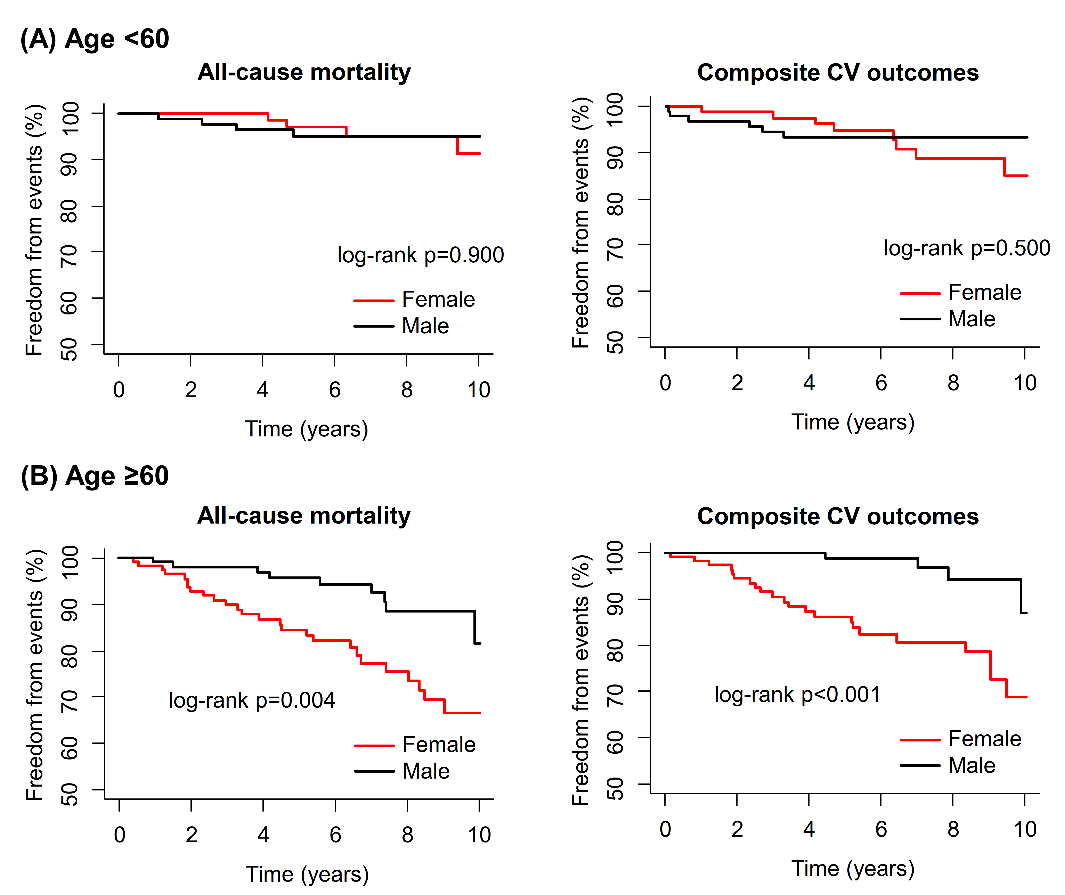
**

**S7 Fig. Sex differences of event-free survival curves for all-cause mortality and composite cardiovascular outcomes, stratified by age of 60 years, after propensity score matching**

Results were consistent with those of the original HCM cohort.

**Supplementary Tables**

**S1 Table. Incidence rates of clinical outcomes according to sex**

|  | Total | Women | IR^*^ (95% CI) | Men | IR^*^ (95% CI) |
| --- | --- | --- | --- | --- | --- |
| All-cause death | 64 (7.7) | 32 (14.3) | 2.33 (1.52-3.14) | 32 (5.2) | 0.81 (0.53-1.09) |
| Cardiovascular death | 32 (3.8) | 21 (9.4) | 1.53 (0.87-2.18) | 11 (1.8) | 0.28 (0.11-0.44) |
| Non-cardiovascular death | 32 (3.8) | 11 (4.9) | 0.80 (0.33-1.27) | 21 (3.4) | 0.53 (0.30-0.76) |
| SCD/SCD equivalent events | 22 (2.6) | 8 (3.6) | 0.58 (0.18-0.99) | 14 (2.3) | 0.36 (0.17-0.54) |
| HF events^†^ | 58 (6.9) | 29 (13.0) | 2.22 (1.41-3.03) | 29 (4.7) | 0.75 (0.48-1.02) |
| Composite cardiovascular outcomes^‡^ | 54 (6.5) | 31 (13.9) | 2.29 (1.49-3.10) | 23 (3.8) | 0.59 (0.35-0.83) |

^*^Incidence rates per 100 person-years

^†^HF events = Admission for HF + Heart transplantation + Death due to HF + Progression to New York Heart Association functional class III/IV
There were 20 patients with admission due to HF, 7 with HF-related death, 2 with heart transplantation due to end-stage HF, and 39 with progression to NYHA III/IV.

^‡^Composite cardiovascular outcomes = Cardiovascular death + SCD/SCD equivalent events + Admission for HF + Heart transplantation

HF, heart failure; ICD, implantable cardioverter-defibrillator; SCD, sudden cardiac death.

**S2 Table. Univariable clinical predictors of all-cause mortality and composite cardiovascular outcomes**

|  | All-cause mortality | | Composite cardiovascular outcomes | |
| --- | --- | --- | --- | --- |
|  | HR (95% CI) | p-value | HR (95% CI) | p-value |
| Female sex | 2.87 (1.76-4.68) | <0.001 | 3.89 (2.27-6.66) | <0.001 |
| Age (year) | 1.07 (1.04-1.10) | <0.001 | 1.03 (1.00-1.06) | 0.025 |
| Body mass index (kg/m^2^) | 0.86 (0.79-0.94) | 0.001 | 0.98 (0.89-1.07) | 0.581 |
| NYHA class (per 1-class increase) | 1.82 (1.24-2.67) | 0.002 | 2.43 (1.64-3.61) | <0.001 |
| Hypertension | 1.43 (0.88-2.34) | 0.151 | 1.17 (0.69-2.00) | 0.564 |
| Diabetes mellitus | 1.26 (0.70-2.28) | 0.447 | 1.14 (0.59-2.21) | 0.698 |
| Chronic kidney disease | 6.73 (3.20-14.1) | <0.001 | 2.79 (0.87-8.94) | 0.085 |
| Liver disease | 2.64 (1.31-5.35) | 0.007 | 0.95 (0.30-3.04) | 0.931 |
| Ischemic heart disease | 1.40 (0.75-2.63) | 0.290 | 0.92 (0.42-2.04) | 0.839 |
| Atrial fibrillation | 2.50 (1.43-4.35) | 0.001 | 4.45 (2.57-7.69) | <0.001 |
| Stroke | 2.91 (1.58-5.35) | 0.001 | 2.82 (1.45-5.48) | 0.002 |
| Baseline medication |  |  |  |  |
| Use of oral anticoagulants | 2.73 (1.46-5.11) | 0.002 | 3.42 (1.80-6.51) | <0.001 |
| Use of beta-blockers | 1.59 (0.97-2.60) | 0.067 | 1.60 (0.94-2.74) | 0.085 |
| Use of calcium channel blockers (non-dihydropyridine) | 0.98 (0.48-1.97) | 0.946 | 1.04 (0.49-2.21) | 0.911 |
| Use of calcium channel blockers (dihydropyridine) | 1.80 (0.98-3.30) | 0.060 | 1.20 (0.57-2.54) | 0.638 |
| Use of ACE inhibitors/ARBs | 1.81 (1.09-3.01) | 0.021 | 1.51 (0.86-2.66) | 0.153 |
| Use of diuretics | 3.60 (2.04-6.33) | <0.001 | 5.45 (3.09-9.61) | <0.001 |
| SCD risk score (%) | 1.02 (0.93-1.13) | 0.618 | 1.16 (1.09-1.23) | <0.001 |
| Max. LV wall thickness, mm | 1.04 (0.98-1.10) | 0.180 | 1.07 (1.02-1.14) | 0.012 |
| Max. LVOT gradient, per 10 mmHg | 1.06 (1.00-1.14) | 0.059 | 1.09 (1.03-1.16) | 0.006 |
| Left atrial dimension, mm | 1.06 (1.02-1.09) | 0.001 | 1.11 (1.08-1.15) | <0.001 |
| LV ejection fraction (%) | 0.95 (0.92-0.98) | 0.002 | 0.92 (0.89-0.96) | <0.001 |

NYHA, New York Heart Association; SCD, sudden cardiac death.

**S3 Table. Multivariable Cox regression analyses for predictors of clinical outcomes**

|  | All-cause mortality | | CV death | | Non-CV death | |
| --- | --- | --- | --- | --- | --- | --- |
| Variables | HR (95% CI) | p-value | HR (95% CI) | p-value | HR (95% CI) | p-value |
| Female sex | 1.88 (1.11-3.20) | 0.019 | 4.56 (2.10-9.91) | <0.001 | 0.73 (0.33-1.62) | 0.437 |
| Age (year) | 1.06 (1.03-1.09) | <0.001 | 1.04 (1.00-1.07) | 0.043 | 1.09 (1.05-1.14) | <0.001 |
| Body mass index (kg/m^2^) | 0.91 (0.84-0.99) | 0.036 | 1.03 (0.92-1.14) | 0.613 | 0.80 (0.70-0.91) | <0.001 |
| NYHA class (per 1-class) | 1.27 (0.83-1.93) | 0.276 | 1.45 (0.81-2.59) | 0.210 | 1.11 (0.59-2.08) | 0.741 |
| SCD risk score (%) | 1.09 (0.98-1.20) | 0.111 | 1.12 (0.99-1.28) | 0.077 | 1.05 (0.88-1.24) | 0.603 |
| LV ejection fraction (%) | 0.95 (0.92-0.98) | 0.003 | 0.92 (0.88-0.96) | <0.001 | 0.99 (0.95-1.04) | 0.778 |
|  | SCD/SCD equivalent events | | HF events | | Composite CV outcomes | |
| Variables | HR (95% CI) | p-value | HR (95% CI) | p-value | HR (95% CI) | p-value |
| Female sex | 2.19 (0.84-5.69) | 0.107 | 2.10 (1.20-3.66) | 0.009 | 3.60 (2.00-6.49) | <0.001 |
| Age (year) | 1.01 (0.97-1.05) | 0.675 | 1.02 (0.99-1.04) | 0.171 | 1.03 (1.00-1.05) | 0.041 |
| Body mass index (kg/m^2^) | 1.14 (1.00-1.29) | 0.048 | 0.99 (0.90-1.08) | 0.749 | 1.04 (0.95-1.13) | 0.499 |
| NYHA class (per 1-class) | 1.33 (0.66-2.69) | 0.423 | 2.67 (1.78-4.01) | <0.001 | 1.76 (1.14-2.72) | 0.011 |
| SCD risk score (%) | 1.28 (1.18-1.38) | <0.001 | 1.10 (1.01-1.21) | 0.032 | 1.22 (1.14-1.31) | <0.001 |
| LV ejection fraction (%) | 0.93 (0.88-0.98) | 0.013 | 0.93 (0.90-0.96) | <0.001 | 0.92 (0.89-0.96) | <0.001 |

CV, cardiovascular; ICD, implantable cardioverter defibrillator; HF, heart failure; NYHA, New York Heart Association; SCD, sudden cardiac death.

**S4 Table. Multivariable Cox regression analyses for predictors of clinical outcomes, in subset of patients with cardiac magnetic resonance imaging (n=752)**

|  | All-cause mortality | | CV death | | Non-CV death | |
| --- | --- | --- | --- | --- | --- | --- |
| Variables | HR (95% CI) | p-value | HR (95% CI) | p-value | HR (95% CI) | p-value |
| Female sex | 3.03 (1.60-5.74) | <0.001 | 6.97 (2.61-18.6) | <0.001 | 1.50 (0.61-3.66) | 0.376 |
| Age (year) | 1.05 (1.02-1.09) | <0.001 | 1.03 (0.99-1.07) | 0.139 | 1.10 (1.04-1.16) | <0.001 |
| Body mass index (kg/m^2^) | 0.94 (0.85-1.04) | 0.219 | 1.04 (0.91-1.18) | 0.578 | 0.86 (0.74-0.99) | 0.041 |
| NYHA class (per 1-class) | 1.41 (0.88-2.28) | 0.156 | 1.86 (0.98-3.54) | 0.058 | 1.02 (0.50-2.09) | 0.964 |
| SCD risk score (%) | 0.99 (0.85-1.16) | 0.935 | 1.08 (0.91-1.27) | 0.371 | 0.85 (0.61-1.19) | 0.343 |
| LV ejection fraction (%) | 0.96 (0.93-1.00) | 0.055 | 0.93 (0.88-0.98) | 0.009 | 1.00 (0.96-1.04) | 0.880 |
| Extensive LGE^*^ | 2.61 (1.37-4.97) | 0.003 | 3.64 (1.45-9.16) | 0.006 | 2.04 (0.80-5.22) | 0.135 |
|  | SCD/SCD equivalent events | | HF events | | Composite CV outcomes | |
| Variables | HR (95% CI) | p-value | HR (95% CI) | p-value | HR (95% CI) | p-value |
| Female sex | 2.56 (0.89-7.36) | 0.081 | 2.52 (1.35-4.67) | 0.003 | 4.67 (2.29-9.52) | <0.001 |
| Age (year) | 1.01 (0.97-1.05) | 0.509 | 1.02 (0.99-1.04) | 0.147 | 1.02 (0.99-1.04) | 0.211 |
| Body mass index (kg/m^2^) | 1.08 (0.93-1.25) | 0.320 | 1.02 (0.93-1.12) | 0.701 | 1.04 (0.94-1.14) | 0.461 |
| NYHA class (per 1-class) | 1.41 (0.68-2.91) | 0.356 | 2.55 (1.67-3.90) | <0.001 | 2.16 (1.35-3.45) | 0.001 |
| SCD risk score (%) | 1.25 (1.15-1.36) | <0.001 | 1.08 (0.98-1.18) | 0.126 | 1.21 (1.12-1.30) | <0.001 |
| LV ejection fraction (%) | 0.95 (0.89-1.01) | 0.110 | 0.92 (0.89-0.96) | <0.001 | 0.93 (0.90-0.97) | <0.001 |
| Extensive LGE^*^ | 2.38 (0.84-6.70) | 0.102 | 2.96 (1.60-5.47) | <0.001 | 2.62 (1.31-5.26) | 0.007 |

^*^Defined as either estimated to be extensive and diffuse by visual inspection or comprising ≥15% of LV mass on quantification

CV, cardiovascular; ICD, implantable cardioverter defibrillator; HF, heart failure; NYHA, New York Heart Association; SCD, sudden cardiac death.

**S5 Table. Clinical outcomes according to sex and age strata**

|  | Age <60 |  | Age ≥60 |  |
| --- | --- | --- | --- | --- |
|  | Women | Men | Women | Men |
| Number of patients | 89 | 395 | 134 | 217 |
| *Number of events* | | | | |
| All-cause mortality | 4 (4.5) | 16 (4.1) | 28 (20.9) | 16 (7.4) |
| Cardiovascular death | 4 (4.5) | 7 (1.8) | 17 (12.7) | 4 (1.8) |
| Non-cardiovascular death | 0 (0) | 9 (2.3) | 11 (8.2) | 12 (5.5) |
| SCD/SCD equivalent events | 2 (2.2) | 12 (3.0) | 6 (4.5) | 2 (0.9) |
| HF events^*^ | 7 (7.9) | 18 (4.6) | 22 (16.4) | 11 (5.1) |
| Composite cardiovascular outcomes^†^ | 9 (9.0) | 15 (3.8) | 23 (17.2) | 8 *3.7) |
| *Incidence rates per 100 person-years (95% CI)* | | | | |
| All-cause mortality | 0.66 (0.01-1.32) | 0.62 (0.32-0.93) | 3.63 (2.28-4.97) | 1.15 (0.59-1.72) |
| Composite cardiovascular outcomes^†^ | 1.35 (0.41-2.29) | 0.59 (0.29-0.89) | 3.03 (1.79-4.27) | 0.58 (0.18-0.98) |

^*^HF events = Admission for HF + Heart transplantation + Death due to HF + Progression to New York Heart Association functional class III/IV
^†^Composite cardiovascular outcomes = Cardiovascular death + SCD/SCD equivalent events + Admission for HF + Heart transplantation

HF, heart failure; ICD, implantable cardioverter-defibrillator; SCD, sudden cardiac death.

**S6 Table. Baseline clinical and echocardiographic characteristics of the age-matched cohort**

|  | Women (n=218) | Men (n=218) | p-value |
| --- | --- | --- | --- |
| Age, years | 60.1 ± 12.8 | 59.9 ± 12.5 | 0.915 |
| BMI, kg/m^2^ | 24.4 ± 3.6 | 25.0 ± 2.8 | 0.069 |
| Family history of HCM | 24 (11.0) | 12 (5.5) | 0.067 |
| Family history of SCD | 36 (16.5) | 25 (11.5) | 0.167 |
| Non-sustained ventricular tachycardia | 43 (21.3) | 48 (23.5) | 0.904 |
| Syncope | 34 (15.6) | 28 (12.8) | 0.493 |
| NYHA class III-IV | 18 (8.3) | 6 (2.8) | 0.021 |
| 5-year SCD risk score (%) | 2.6 ± 1.7 | 2.5 ± 2.2 | 0.617 |
| Hypertension | 97 (44.5) | 102 (46.8) | 0.701 |
| Diabetes mellitus | 39 (17.9) | 45 (20.6) | 0.544 |
| Chronic kidney disease | 5 (2.3) | 9 (4.1) | 0.415 |
| Liver disease | 7 (3.2) | 12 (5.5) | 0.348 |
| Ischemic heart disease | 28 (12.8) | 32 (14.7) | 0.677 |
| Atrial fibrillation | 39 (17.9) | 45 (20.6) | 0.544 |
| Stroke | 23 (10.6) | 26 (11.9) | 0.762 |
| Baseline medication |  |  |  |
| Use of oral anticoagulants | 15 (6.9) | 18 (8.3) | 0.717 |
| Use of beta-blockers | 87 (39.9) | 75 (34.4) | 0.276 |
| Use of calcium channel blockers (non-dihydropyridine) | 39 (17.9) | 34 (15.6) | 0.608 |
| Use of calcium channel blockers (dihydropyridine) | 25 (11.5) | 37 (17.0) | 0.131 |
| Use of ACE inhibitors/ARBs | 58 (26.6) | 59 (27.1) | >0.999 |
| Use of diuretics | 35 (16.1) | 22 (10.1) | 0.088 |
| Echocardiographic data |  |  |  |
| LV end-diastolic dimension, mm | 45.6 ± 5.2 | 48.7 ± 5.1 | <0.001 |
| Indexed LVEDD, mm/m^2^ | 29.0 ± 4.0 | 26.9 ± 3.1 | <0.001 |
| LV end-systolic dimension, mm | 27.1 ± 4.3 | 29.0 ± 4.1 | <0.001 |
| Indexed LVESD, mm/m^2^ | 17.2 ± 3.2 | 16.0 ± 2.4 | <0.001 |
| LV ejection fraction (%) | 64.5 ± 7.4 | 64.4 ± 6.4 | 0.853 |
| LV ejection fraction <50% | 6 (2.8) | 3 (1.4) | 0.501 |
| LA dimension, mm | 44.1 ± 7.1 | 44.3 ± 6.9 | 0.735 |
| E, m/s | 0.65 ± 0.21 | 0.63 ± 0.22 | 0.218 |
| e', cm/s | 4.6 ± 1.9 | 5.1 ± 1.8 | 0.007 |
| s', cm/s | 6.1 ± 1.4 | 6.5 ± 1.5 | 0.024 |
| E/e' ratio | 15.9 ± 7.4 | 13.4 ± 6.4 | <0.001 |
| Pulmonary artery systolic pressure, mmHg | 33.6 ± 7.8 | 32.0 ± 6.4 | 0.173 |
| Maximum LV wall thickness, mm | 17.1 (15.5-20.0) | 17.0 (15.2-20.0) | 0.759 |
| Indexed max. LV wall thickness, mm/m^2^ | 11.1 (9.9-13.0) | 9.7 (8.5-11.1) | <0.001 |
| LV mass index, g/m^2^ | 131 ± 48 | 129 ± 44 | 0.710 |
| Maximum LVOT gradient ≥30mmHg | 41 (18.8) | 29 (13.3) | 0.151 |
| Max. LVOT gradient, mmHg (in obstructive HCM patients) | 82 (57-117) | 54 (42-92) | 0.012 |
| LV-GLS (%) | -16.7 ± 4.8 | -14.8 ± 4.4 | <0.001 |

DT, deceleration time; E, peak early diastolic mitral inflow velocity; e', early diastolic mitral annular velocity; HCM, hypertrophic cardiomyopathy; LV, left ventricular; LV-GLS, LV global longitudinal strain; LVOT, LV outflow tract; NYHA, New York Heart Association; SCD, sudden cardiac death; s’, systolic mitral annular velocity.

**S7 Table. Clinical outcomes in the age-matched cohort**

|  | Total | Women | Men |
| --- | --- | --- | --- |
| Number of patients | 436 | 218 | 218 |
| All-cause mortality | 45 (10.3) | 30 (13.8) | 15 (6.9) |
| Cardiovascular death | 23 (5.3) | 19 (8.7) | 4 (1.8) |
| Non-cardiovascular death | 22 (5.0) | 11 (5.0) | 11 (5.0) |
| SCD/SCD equivalent events | 12 (2.8) | 8 (3.7) | 4 (1.8) |
| HF events^*^ | 37 (8.5) | 28 (12.8) | 9 (4.1) |
| Composite cardiovascular outcomes^†^ | 38 (8.7) | 29 (13.3) | 9 (4.1) |

^*^HF events = Admission for HF + Heart transplantation + Death due to HF + Progression to New York Heart Association functional class III/IV
^†^Composite cardiovascular outcomes = Cardiovascular death + SCD/SCD equivalent events + Admission for HF + Heart transplantation

HF, heart failure; ICD, implantable cardioverter-defibrillator; SCD, sudden cardiac death.

**S8 Table. Absolute standardized difference (ASD) before and after propensity score matching**

|  | Before propensity score matching | | | After propensity score matching | | |
| --- | --- | --- | --- | --- | --- | --- |
|  | Women (n=223) | Men (n=612) | ASD | Women (n=209) | Men (n=209) | ASD |
| Age, years | 59.9 ± 13.5 | 54.9 ± 11.4 | 0.398^*^ | 59.2 ± 13.6 | 59.2 ± 10.8 | 0.001 |
| BMI, kg/m^2^ | 24.4 ± 3.6 | 25.4 ± 2.8 | 0.317^*^ | 24.4 ± 3.7 | 24.5 ± 2.6 | 0.034 |
| Family history of HCM | 25 (11.2) | 52 (8.5) | 0.091 | 23 (11.0) | 28 (13.4) | 0.073 |
| Family history of SCD | 36 (16.1) | 71 (11.6) | 0.131^*^ | 31 (14.8) | 39 (18.7) | 0.102^*^ |
| NSVT | 44 (19.7) | 110 (18.0) | 0.045 | 40 (19.1) | 46 (22.0) | 0.071 |
| Syncope | 36 (16.1) | 80 (13.1) | 0.087 | 36 (17.2) | 33 (15.8) | 0.039 |
| NYHA class III-IV | 18 (8.1) | 12 (2.0) | 0.282^*^ | 8 (3.8) | 10 (4.8) | 0.047 |
| 5-year SCD risk score (%) | 2.6 ± 1.8 | 2.7 ± 2.5 | 0.058 | 2.6 ± 1.8 | 2.7 ± 2.6 | 0.039 |
| Hypertension | 99 (44.4) | 252 (41.2) | 0.065 | 92 (44.0) | 89 (42.6) | 0.029 |
| Diabetes mellitus | 39 (17.5) | 103 (16.8) | 0.017 | 37 (17.7) | 29 (13.9) | 0.105^*^ |
| Chronic kidney disease | 5 (2.2) | 13 (2.1) | 0.008 | 5 (2.4) | 5 (2.4) | <0.001 |
| Liver disease | 8 (3.6) | 38 (6.2) | 0.122^*^ | 8 (3.8) | 11 (5.3) | 0.069 |
| Ischemic heart disease | 28 (12.6) | 78 (12.7) | 0.006 | 28 (13.4) | 26 (12.4) | 0.028 |
| Atrial fibrillation | 29 (13.0) | 81 (13.2) | 0.007 | 27 (12.9) | 32 (15.3) | 0.069 |
| Stroke | 17 (7.6) | 57 (9.3) | 0.061 | 17 (8.1) | 16 (7.7) | 0.018 |
| Use of oral anticoagulants | 15 (6.7) | 48 (7.8) | 0.043 | 14 (6.7) | 17 (8.1) | 0.055 |
| Use of beta-blockers | 88 (39.5) | 198 (32.4) | 0.148^*^ | 82 (39.2) | 78 (37.3) | 0.039 |
| Use of calcium channel blockers (non-dihydropyridine) | 40 (17.9) | 73 (11.9) | 0.169^*^ | 38 (18.2) | 31 (14.8) | 0.090 |
| Use of calcium channel blockers (dihydropyridine) | 27 (12.1) | 77 (12.6) | 0.014 | 25 (12.0) | 31 (14.8) | 0.084 |
| Use of ACE inhibitors/ARBs | 59 (26.5) | 138 (22.5) | 0.091 | 54 (25.8) | 50 (23.9) | 0.044 |
| Use of diuretics | 35 (15.7) | 46 (7.5) | 0.257^*^ | 31 (14.8) | 20 (9.6) | 0.161^*^ |

HCM, hypertrophic cardiomyopathy; NYHA, New York Heart Association; NSVT, Non-sustained ventricular tachycardia; SCD, sudden cardiac death.
ASD <0.10~0.20 implies good balance between the two groups. The asterisk (^*^) denotes variables with ASD ≥0.10.

**S9 Table. Baseline echocardiographic characteristics after propensity score matching**

|  | Women (n=218) | Men (n=218) | p-value |
| --- | --- | --- | --- |
| LVEDD, mm | 45.7 ± 4.9 | 48.1 ± 5.2 | <0.001 |
| Indexed LVEDD, mm/m^2^ | 29.0 ± 3.9 | 26.8 ± 3.2 | <0.001 |
| LVESD, mm | 27.1 ± 4.3 | 28.9 ± 4.5 | <0.001 |
| Indexed LVESD, mm/m^2^ | 17.2 ± 3.2 | 16.1 ± 2.6 | <0.001 |
| LV ejection fraction (%) | 64.5 ± 7.4 | 63.6 ± 7.0 | 0.164 |
| LV ejection fraction <50% | 6 (2.7) | 5 (2.4) | >0.999 |
| Left atrial dimension, mm | 44.0 ± 7.1 | 44.2 ± 6.9 | 0.770 |
| E, m/s | 0.65 ± 0.21 | 0.60 ± 0.18 | 0.010 |
| e', cm/s | 4.6 ± 1.9 | 5.2 ± 1.6 | 0.001 |
| s', cm/s | 6.1 ± 1.4 | 6.4 ± 1.5 | 0.091 |
| E/e' ratio | 16.0 ± 7.5 | 12.3 ± 4.8 | <0.001 |
| Pulmonary artery systolic pressure, mmHg | 33.4 ± 6.9 | 31.0 ± 6.2 | 0.032 |
| Max. LV wall thickness, mm | 17.5 (15.6-20.3) | 17.0 (15.3-19.6) | 0.192 |
| Indexed max. LV wall thickness, mm/m^2^ | 11.2 (9.9-13.1) | 9.6 (8.5-11.0) | <0.001 |
| Max. LVOT gradient ≥30mmHg | 40 (19.1) | 24 (11.5) | 0.042 |
| Max. LVOT gradient, mmHg (in obstructive HCM patients) | 87 (56-116) | 52 (38-88) | 0.012 |
| LV-GLS (%) | -16.7 ± 4.9 | -14.5 ± 4.3 | <0.001 |

E, peak early diastolic mitral inflow velocity; e', early diastolic mitral annular velocity; HCM, hypertrophic cardiomyopathy; LV, left ventricular; LV-GLS, LV global longitudinal strain; LVOT, LV outflow tract.

**S10 Table. Clinical outcomes after propensity score matching**

|  | Total | Women | Men |
| --- | --- | --- | --- |
| Number of patients | 418 | 209 | 209 |
| All-cause mortality | 43 (10.3) | 30 (14.4) | 13 (6.2) |
| Cardiovascular death | 24 (5.7) | 20 (9.6) | 4 (1.9) |
| Non-cardiovascular death | 19 (4.5) | 10 (4.8) | 9 (4.3) |
| SCD/SCD equivalent events | 12 (2.9) | 8 (3.8) | 4 (1.9) |
| HF events^*^ | 38 (9.1) | 26 (12.4) | 12 (5.7) |
| Composite cardiovascular outcomes^†^ | 40 (9.6) | 30 (14.4) | 10 (4.8) |

^*^HF events = Admission for HF + Heart transplantation + Death due to HF + Progression to New York Heart Association functional class III/IV
^†^Composite cardiovascular outcomes = Cardiovascular death + SCD/SCD equivalent events + Admission for HF + Heart transplantation

HF, heart failure; ICD, implantable cardioverter-defibrillator; SCD, sudden cardiac death.

**Supplementary References**

1. Lang RM, Badano LP, Mor-Avi V, Afilalo J, Armstrong A, Ernande L, et al. Recommendations for cardiac chamber quantification by echocardiography in adults: an update from the American Society of Echocardiography and the European Association of Cardiovascular Imaging. J Am Soc Echocardiogr. 2015;28(1):1-39 e14. Epub 2015/01/07. doi: 10.1016/j.echo.2014.10.003. PubMed PMID: 25559473.

2. Choi HM, Kim KH, Lee JM, Yoon YE, Lee SP, Park EA, et al. Myocardial fibrosis progression on cardiac magnetic resonance in hypertrophic cardiomyopathy. Heart. 2015;101(11):870-6. Epub 2015/04/22. doi: 10.1136/heartjnl-2014-306555. PubMed PMID: 25897040.

3. Park YJ, Park SJ, Kim EK, Park KM, Lee SC, On YK, et al. Semi-quantitative versus quantitative assessments of late gadolinium enhancement extent for predicting spontaneous ventricular tachyarrhythmia events in patients with hypertrophic cardiomyopathy. Sci Rep. 2020;10(1):2920. Epub 2020/02/23. doi: 10.1038/s41598-020-59804-8. PubMed PMID: 32076039; PubMed Central PMCID: PMCPMC7031259.

4. Ommen SR, Mital S, Burke MA, Day SM, Deswal A, Elliott P, et al. 2020 AHA/ACC Guideline for the Diagnosis and Treatment of Patients With Hypertrophic Cardiomyopathy: A Report of the American College of Cardiology/American Heart Association Joint Committee on Clinical Practice Guidelines. Circulation. 2020;142(25):e558-e631. Epub 2020/11/21. doi: 10.1161/CIR.0000000000000937. PubMed PMID: 33215931.
